# Spatial Distribution of Black Bear Incident Reports in Michigan

**DOI:** 10.1371/journal.pone.0154474

**Published:** 2016-04-27

**Authors:** Jamie E. McFadden-Hiller, Dean E. Beyer, Jerrold L. Belant

**Affiliations:** 1 Carnivore Ecology Laboratory, Forest and Wildlife Research Center, Mississippi State University, Starkville, Mississippi, United States of America; 2 Wildlife Division, Michigan Department of Natural Resources, Marquette, Michigan, United States of America; Institute of Agronomy, University of Lisbon, PORTUGAL

## Abstract

Interactions between humans and carnivores have existed for centuries due to competition for food and space. American black bears are increasing in abundance and populations are expanding geographically in many portions of its range, including areas that are also increasing in human density, often resulting in associated increases in human-bear conflict (hereafter, bear incidents). We used public reports of bear incidents in Michigan, USA, from 2003–2011 to assess the relative contributions of ecological and anthropogenic variables in explaining the spatial distribution of bear incidents and estimated the potential risk of bear incidents. We used weighted Normalized Difference Vegetation Index mean as an index of primary productivity, region (i.e., Upper Peninsula or Lower Peninsula), primary and secondary road densities, and percentage land cover type within 6.5-km^2^ circular buffers around bear incidents and random points. We developed 22 *a priori* models and used generalized linear models and Akaike’s Information Criterion (AIC) to rank models. The global model was the best compromise between model complexity and model fit (*w* = 0.99), with a ΔAIC 8.99 units from the second best performing model. We found that as deciduous forest cover increased, the probability of bear incident occurrence increased. Among the measured anthropogenic variables, cultivated crops and primary roads were the most important in our AIC-best model and were both positively related to the probability of bear incident occurrence. The spatial distribution of relative bear incident risk varied markedly throughout Michigan. Forest cover fragmented with agriculture and other anthropogenic activities presents an environment that likely facilitates bear incidents. Our map can help wildlife managers identify areas of bear incident occurrence, which in turn can be used to help develop strategies aimed at reducing incidents. Researchers and wildlife managers can use similar mapping techniques to assess locations of specific conflict types or to address human impacts on endangered species.

## Introduction

Interactions between humans and carnivores have existed for centuries due to competition for food and space [1]. These interactions have increased over time and have largely involved variables that can be categorized into human health and safety, economical gains and losses (e.g., revenue from hunting, compensation for agricultural damage), and ecological concerns (e.g., destruction of habitat, collapse of wildlife populations; [2]). The re-establishment of large carnivores on some landscapes since the 1960s (e.g., [3, 4]) is due in part to improved human attitudes towards some carnivore species [5]. However, highly variable and often negative or indifferent public perceptions remain for large carnivore species (e.g., cougars [*Puma concolor*] and black bears [*Ursus americanus*]; [6, 7]), making population recovery and promoting human-wildlife coexistence challenging for managers. Regardless of public perceptions, black bears, specifically, are increasing in abundance and populations are expanding geographically in many portions of its range [8, 9]. With increasing human and bear populations in areas with intersecting anthropogenic (e.g., agriculture, residential development) and ecological variables (e.g., land cover type, vegetation productivity), human-black bear interactions have increased [10, 11], and are primarily related to availability of anthropogenic food (e.g., agricultural crops, human refuse; [12, 13]).

Human-wildlife interactions often increase during intervals of scarce natural foods when wildlife may use potentially more abundant and accessible anthropogenic food sources [14]. Bears are opportunistic foragers and during extended periods of low natural food availability may increase consumption of anthropogenic foods including agricultural crops, apiaries, bird feed, human refuse, and pet and livestock foods [15–17]. Such shifts in foraging behaviors may originate from individual predation avoidance or interference competition (i.e., the despotic distribution hypothesis; [18]). Regardless of the proximate cause, these foraging behaviors can lead to human-bear interactions ranging in severity from property damage and consumption of anthropogenic foods to vehicle collisions and human safety concerns [19–21]. While damage caused by black bears may be limited compared to other wildlife species, individual landowners can incur substantial costs [22].

Black bears are considered a forest obligate species [23] but can persist in highly fragmented areas, especially where suitable habitat, such as forested riparian zones, is present [24, 25]. However, as landscape heterogeneity increases causing alterations in the distribution and continuity of preferred habitat and resources, bears may increase their space use to meet biological demands [18, 26]. Increases in human-wildlife interactions often result from increased space use by large carnivores in fragmented landscapes to obtain sufficient resources [27–29].

Human infrastructure, such as roads, fragment landscapes and can substantially affect human-wildlife interactions [30]. Because large carnivore species exhibit a variety of positive (e.g., increased reproductive success) or negative responses (e.g., decreased survival) to roads and maintain large home ranges, they not only have many opportunities to interact with humans but may also be particularly sensitive to those interactions [31, 32, 12]. For black bears, road type (e.g., main vs. tertiary roads), traffic volume, and primary use of road (e.g., hunter access; [33, 34]) can affect bear use, resulting in roads serving as travel corridors positively affecting survival and reproduction or as semipermeable movement barriers with increased mortality risk from vehicle collisions and loss of habitat through disturbance [28].

We assessed the relative contributions of ecological and anthropogenic variables in explaining variation in the spatial distribution of publically reported black bear incidents (e.g., property damage, crop damage, vehicle-bear collisions; hereafter, bear incident reports) and estimated the probability of bear incident report occurrence in Michigan, USA. We expected more bear incident reports in areas with lesser natural food availability (based on an index of vegetation productivity) and greater road densities. We also expected areas with greater percentages of agriculture land cover located near forested areas to have more bear incident reports. Rural and suburban development has increased during the last several decades in Michigan, particularly a northern expansion of its residents into areas traditionally containing greater densities of bears [35, 36]. We expected more bear incident reports in portions of the bear population range with increasing rural and suburban development.

## Study Area

**Fig 1:**
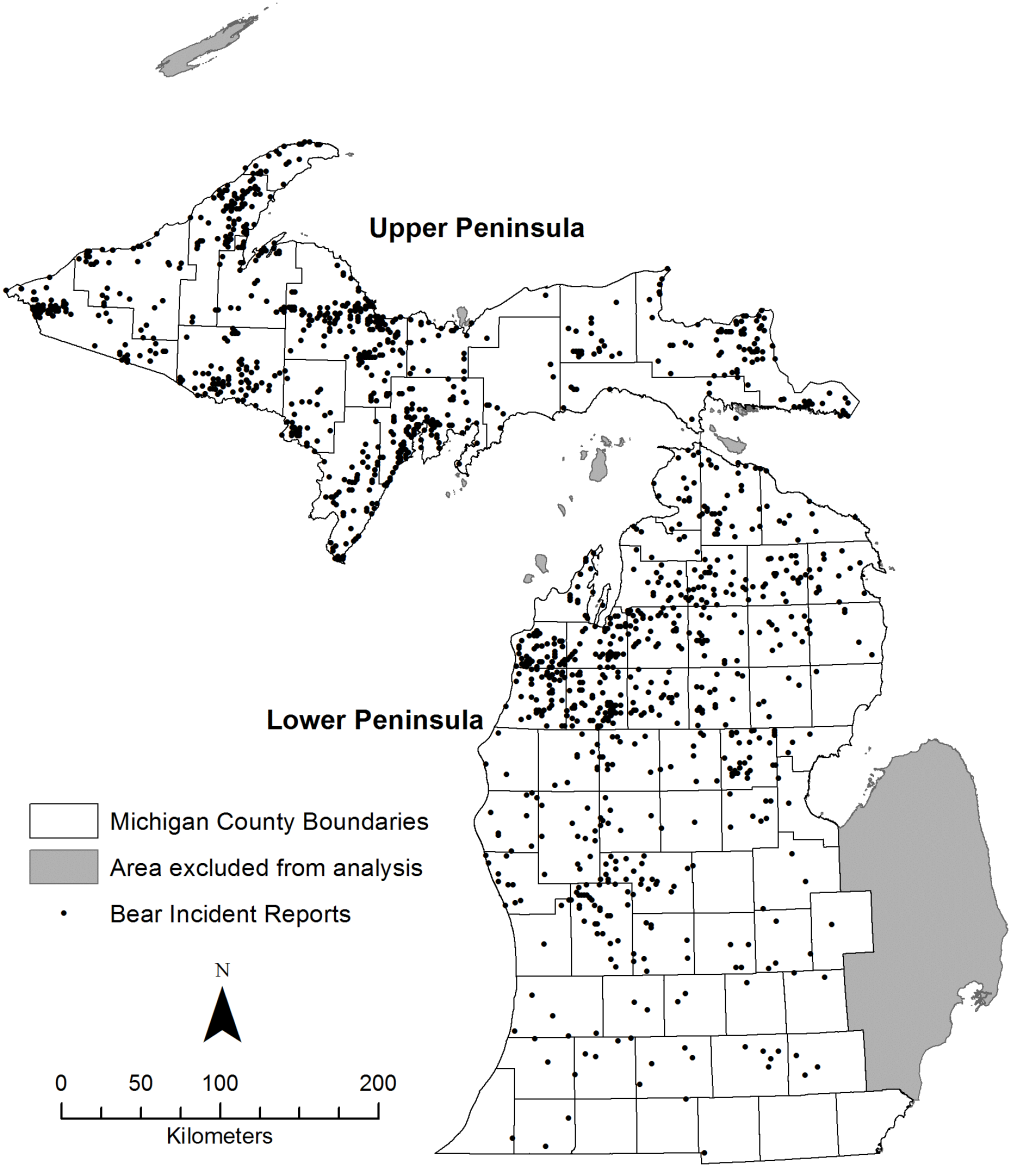
Locations of black bear incident reports in Michigan. Locations at the section level of publically reported black bear incidents (black dots) received by Michigan Department of Natural Resources, Michigan, USA, 2003–2011. Gray areas were excluded from analyses as they contained no black bear incident reports and are outside the black bear range.

Our study area (134,124 km^2^) comprised the Michigan mainland (i.e., excluding islands such as Isle Royale and Mackinac Island) except counties in east-central Michigan as no bear incidents were reported there and they are outside the black bear population range (Fig 1). Our study area contained a human population of 5.66 million [37, 38] with 5.5% (7.2 people/km^2^) residing in the Upper Peninsula (UP; 43,029 km^2^) of Michigan which comprised 32% of the study area. The UP is 45% (19,266 km^2^) publically owned [39] and primarily forested with northern hardwoods and conifers interspersed with agriculture in the southeastern portion [40]. Deciduous forest (33.3%) was the dominant land cover for the region. Topography consists of rolling hills ranging in elevation from 184 to 604 m (mean sea level) in the western portion of the UP to primarily flat and poorly drained peat lands and conifer swamps in the east [40]. Road density in the UP was 0.65 km/km^2^ (28,109 km; [41]).

Human densities, area of agricultural land, and road densities were greater in the Lower Peninsula (LP; 91,095 km^2^), which contained 94.5% of the state’s residents (58.7 people/km^2^; [37]) and is 16% (14,430 km^2^) publically owned [42]. Primary land use included logging interspersed with local farming in the northern hardwood and pine (*Pinus* spp.) forests and widespread agriculture and urban development that replaced much of the oak savannas and hardwood forests in the southern rolling hills and flat lake plains [40]. Cultivated crops (25.6%) was the dominant land cover of the LP. Elevation ranges from 175 to 526 m with some of the highest elevations in the northern portion [40]. Primary and secondary roads occur at a density of 1.69 km/km^2^ (154,058 km^2^; [43]).

The bear population in the UP was estimated at about 7,500 individuals in 1990 [44]. The population fluctuated slightly through the early 2000s and has since increased to almost 8,700 individuals in 2013 [45]. In the northern LP, the population of black bears in 2003 was estimated at about 1,900 individuals [46]. The population has apparently increased slightly to almost 2,000 bears in 2013 [45]. Using 2013 estimates, about 80% of the state’s total black bear population resides in the UP.

## Methods

### Data Collection

In 1994, the Michigan Department of Natural Resources (MDNR) began documenting public reports of bear incidents using a standardized Bear Activity Report form [47]. We obtained reported bear incidents collected in Michigan during 2003–2011 (Fig 1), because the agency began collecting data in electronic format starting in 2003. We excluded bear incidents with incomplete location information and reports documenting only bear sightings because our objective was to model human-bear interaction relationships that resulted in bear incidents (e.g., bear-related property or agriculture damage, pet or livestock attacks, vehicle collisions; [48]). Hereafter, we refer to qualifying reports as bear incident reports. Locations of bear incident reports were recorded at the section level (1 mi^2^; 2.59 km^2^), which consequently served as the spatial scale of our assessment.

We selected 3 times as many random points (i.e., available units) by region to accurately represent available locations within the study area in contrast to bear incident reports (i.e., used units; [49]). For each random point and bear incident report, we assigned a response value of 0 and 1, respectively. We used a 6.5-km^2^ circular buffer centered on the associated section centroid for each bear incident report and on the nearest section centroid for each random point (hereafter, random units). This buffer size was intermediate in size based on daily movements of female and male bears in Michigan (4- and 9-km^2^, respectively; [50]). We obtained eMODIS Normalized Difference Vegetation Index (NDVI) data from 2003–2011 with a spatial resolution of 250-meters and a 16-bit radiometric resolution (i.e., -2,000–10,001 scale; [51]). We used NDVI as an index for the natural sources of vegetative food during the statewide growing season and bear activity (non-hibernation) period (Jun–Sep; [52, 53]). We converted the NDVI data to an 8-bit radiometric resolution (i.e., 0–255 scale); more commonly reported in published literature, estimated the monthly mean values during the growing season, and obtained the seasonal weighted-mean NDVI value for all bear incident reports and random units.

We used spatial data from the National Land Cover Database (NLCD) to estimate the percentage on a continuous scale of each land cover within all bear incident reports and random units [38], and excluded those that contained ≥ 95% water from analyses because bear incident reports cannot occur in open water. Since the open water land cover contained rivers, in addition to lakes, it was included in the model set to account for the biological importance of riparian areas for black bears [25]. Additional land covers from the 2006 NLCD that were included in the analysis were open space development (areas mostly of vegetation with some constructed materials [e.g., parks, large-lot single-family housing units]; impervious surfaces account for < 20% of total cover), high-intensity development (areas where people reside or work in high numbers [e.g., apartment or industrial complexes]; impervious surfaces account for 80–100% of total cover), barren ground (areas of < 15% vegetation cover [e.g., sand dunes, gravel pits]), deciduous forest (areas dominated by trees > 5-m tall that comprise of > 20% of total vegetation cover; > 75% of tree species are deciduous), evergreen forest (areas dominated by trees > 5-m tall that comprise of > 20% of total vegetation cover; > 75% of tree species maintain their leaves year-round [i.e., canopy always has green foliage]), mixed forest (areas dominated by trees > 5-m tall that comprise of > 20% of total vegetation cover; neither deciduous nor evergreen species are > 75% of total tree cover), shrub-scrub (areas dominated by shrubs [e.g., true shrubs, young trees] < 5-m tall with canopy comprised of > 20% of shrubs), grassland-herbaceous (areas with > 80% gramminoid or herbaceous vegetation; not subject to intensive management but can be grazed), pasture-hay (areas with > 20% of grasses, legumes, or grass-legume mixtures planted for seed or hay crop production or livestock grazing), cultivated crops (areas with > 20% crop vegetation cover [e.g., corn, cotton]; includes all actively tilled land), woody wetlands (areas with > 20% forest or shrub land vegetation cover and substrate is periodically saturated or covered with water), and emergent herbaceous wetlands (areas with > 80% perennial herbaceous vegetation cover and substrate is periodically saturated or covered with water).

We classified roads as primary or secondary [41, 54] and estimated the density (km/km^2^) of each road type for each bear incident report and random unit. Primary roads included interstates, highways, and residential roads. Secondary roads included roads that may be paved but have little traffic, including park roads, two-track roads, and vehicular trails. We included region (LP [reference category] or UP) as a covariate to account for biological differences between the two bear populations (e.g., population size and density) since more spatially refined data were not available. We used ArcMap [55], ERDAS Imagine [56], Raster package in Program R [57], Geospatial Modeling Environment [58], and Spatial Analyst Supplemental Tools in ArcGIS for all data extractions.

### Statistical Analyses

To improve model convergence and allow for direct comparisons among independent variables, we centered and scaled independent variables [59]. We used the Pearson product-moment correlation coefficient (*r*) to test for multicollinearity among all continuous independent variables. We assumed multicollinearity did not compromise model results if |*r*| < 0.70 for any pair of independent variables [60]. However, if |*r*| ≥ 0.70 for any pair, we excluded the variable we considered least ecologically important based on literature from analyses. We used generalized linear modeling with logistic regression to assess effects of independent variables on the occurrence of bear incident reports. We assumed that our dependent variable (i.e., occurrence of a bear incident report), from presence-only data, followed a binomial distribution (i.e., conflict vs. no conflict).

We constructed 22 *a priori* models to test our hypotheses regarding the ecological and anthropogenic effects on the occurrence of bear incident reports and grouped models based on our hypotheses (Table 1). We tested for overdispersion by visual inspection of quantile-quantile plots and estimating the variance inflation factor ($\hat{c}$) based on the chi-square goodness-of-fit test [61]. To rank models based on complexity and fit, we used Akaike Information Criterion (AIC; [62]). We used 1^st^ quartiles, medians, and 3^rd^ quartiles to characterize low, medium, and high percentage of land covers and density of roads.

**Table 1:** *A priori* model set.

Hypothesis	Model #	Covariates
Null	1	~ 1
Productivity	2	~ Weighted NDVI^a^ mean
	3	~ Weighted NDVI mean + region^b^
Region	4	~ Region
Anthropogenic Effects	5	~ Primary road density^c^
	6	~ Primary road density + region
	7	~ Secondary road density^d^
	8	~ Secondary road density + region
Habitat	9	~ percent NLCD^e^
	10	~ percent NLCD + region
Productivity & Anthropogenic Effects	11	~ Weighted NDVI mean + primary road density
	12	~ Weighted NDVI mean + primary road density + region
	13	~ Weighted NDVI mean + secondary road density
	14	~ Weighted NDVI mean + secondary road density + region
Productivity & Habitat	15	~ Weighted NDVI mean + percent NLCD
	16	~ Weighted NDVI mean + percent NLCD + region
Anthropogenic Effects & Habitat	17	~ Primary road density + percent NLCD
	18	~ Primary road density + percent NLCD + region
	19	~ Secondary road density + percent NLCD
	20	~ Secondary road density + percent NLCD + region
Productivity & Anthropogenic Effects & Habitat	21	~ Weighted NDVI mean + primary road density + secondary road density + percent NLCD
Global	22	~ Weighted NDVI mean + primary road density + secondary road density + percent NLCD + region

The model set contained 22 additive models with 17 independent variables used in an analysis based on Akaike Information Criterion (AIC) to predict the spatial occurrence of black bear incident reports, Michigan, USA, 2003–2011.

^a^ NDVI = Normalized Difference Vegetation Index.

^b^ Region = regional location in which a given bear incident report occurred (Upper Peninsula or Lower Peninsula).

^c^ Primary road density = interstates, highways, and residential roads.

^d^ Secondary road density = roads that may be paved but have little traffic (e.g., park roads, two-track roads).

^e^; NLCD = National Land Cover Database; percent NLCD—percent area for each land cover (e.g., developed open space, deciduous forest, cultivated crops, etc.).

To evaluate model fit of the AIC-best model, we used an independent data set (i.e., data of bear incident reports collected during 2012–2015). We compared the observed values (bear incident reports) from the independent dataset (fit with a logistic regression for the response variable) with the predicted values (model results) from the AIC-best model using the standard deviation scores (*z*; [61, 63]) with

z = X− μ/σ

where *X* = observed value of bear incident reports, *μ* = predicted value of bear incident reports, and *σ* = standard deviation of values used to estimate probability of bear incident report occurrence from modeling results. We tested for differences between observed and predicted values and assumed no difference existed if *P*>0.05 for the cumulative *P*-value for the *z*-score. We also tested whether the 95% confidence limits (CL) of the slope and intercept of the linear equation of observed versus predicted values included 1 and 0, respectively. We used Program R [64] for all statistical analyses.

## Results

**Fig 2:**
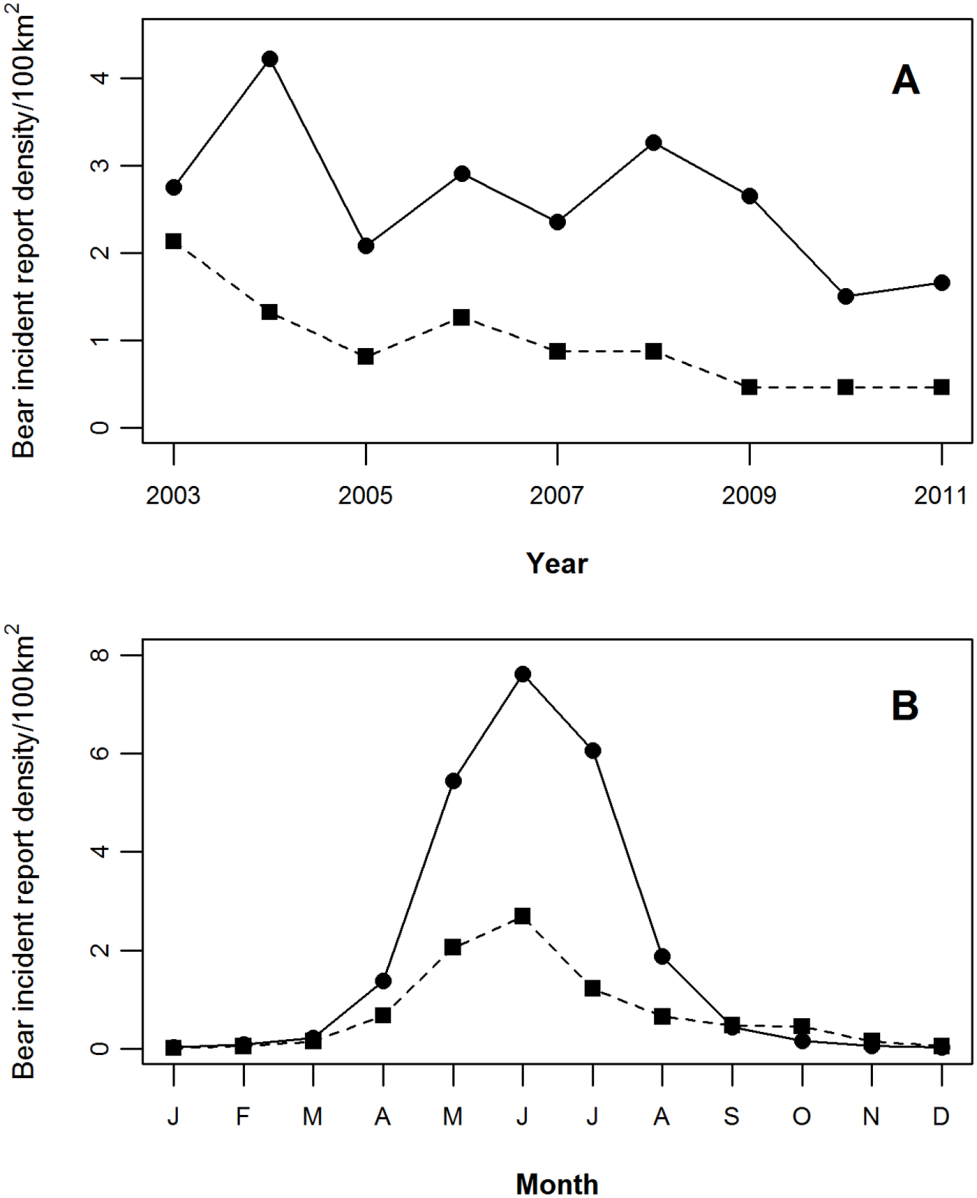
Densities of black bear incident reports in Michigan. Density of black bear incident reports received by Michigan Department of Natural Resources during 2003–2011 for the Upper Peninsula (solid line) and Lower Peninsula (dashed line) regions of the study area with (A) the average annual black bear incident report density and (B) average monthly black bear incident report density.

The MDNR received 2,441 bear incident reports during 2003–2011. We excluded 640 bear incident reports because they lacked adequate location information or were sighting-only reports and 1 bear incident report because the associated buffer contained >95% open water; thus, our final data set contained 1,800 bear incident reports and 5,400 random units (Fig 1; S1 Dataset). On average, the MDNR received 200 (SD = 70.65) bear incident reports annually with about 56% of the bear incident reports occurring in the UP (Fig 2). The LP and UP had annual average bear incident report densities of 0.96/100 km^2^ (95% CL = 0.44–1.48) and 2.60/100 km^2^ (95% CL = 2.26–2.93), respectively. Bear incident reports decreased annually by 0.19/100 km^2^ (95% CL = -0.14–0.51) between 2003 and 2011 (Fig 2A). Bear incident report density peaked during June in both regions with 76% of all reports occurring from May to July (Fig 2B).

Eight pairs of continuous variables were correlated and resulted in the exclusion of 2 NLCD land-covers (low- and medium-intensity development) and human population density. Our global model did not show overdispersion ($\hat{c}$ = 0.99) and residuals showed no lack of fit. The global model was the best compromise between model complexity and model fit (*w* = 0.99), with a ΔAIC 8.99 units from the second best performing model (Table 2). For comparing predicted (model results from the AIC-best model) and observed (bear incident reports from the independent data set) values, our model evaluation yielded a linear equation with a slope of 1.05 (95% confidence limit [CL] = 0.91 to 1.18) and an intercept of −0.07 (95% CL = −0.23 to 0.09; S2 Dataset). The cumulative P-value based on our z-scores was 0.49. Based on our model evaluation procedures, our AIC-best model had acceptable predictive performance.

**Table 2:** Summary of model selection results.

Model	*K*^a^	ΔAIC^b^	*W*^c^
Weighted NDVI^d^ mean + primary road density^e^ + secondary road density^f^ + percent NLCD^g^ + region^h^	18	0.00	0.99
Weighted NDVI mean + primary road density + secondary road density + percent NLCD	17	8.99	0.01
Primary road density + percent NLCD + region	16	29.01	< 0.01
Primary road density + percent NLCD	15	33.61	< 0.01

Akaike Information Criterion (AIC) model selection results for the top 4 models from a set of 22 used to test the spatial relationship between independent variables and the occurrence of black bear incident reports, Michigan, USA, 2003–2011.

^a^ *K* = the number of estimated parameters in the model.

^b^ ΔAIC = AIC difference in relation to the top-ranked model.

^c^ *w* = AIC model weight.

^d^ NDVI = Normalized Difference Vegetation Index.

^e^ Primary road density = interstates, highways, and residential roads.

^f^ Secondary road density = roads that may be paved but have little traffic (e.g., park roads, two-track roads, etc.).

^g^ NLCD = National Land Cover Database; percent NLCD = percent area for each land cover (e.g., developed open space, deciduous forest, cultivated crops, etc.).

^h^ Region = Upper Peninsula or Lower Peninsula.

Deciduous forest, woody wetlands, evergreen forest, open water, mixed forest, grassland-herbaceous, emergent herbaceous wetlands, shrub-scrub, barren land, weighted NDVI mean, cultivated crops, pasture-hay, developed open space, primary road density, secondary road density, and region were positively associated with bear incident reports; the confidence intervals of remaining parameters included zero and were considered insignificant (Table 3). Deciduous forest was the dominant land cover for bear incident reports with an average area percentage of 30.5% (95% CL = 29.0–32.0; Table 4). The relationship between probability of bear incident report occurrence and deciduous forest, cultivated crop, and primary roads was the same for both regions. Specifically, probability of bear incident report occurrence was low where deciduous forest cover was <40%. Among the measured anthropogenic variables, cultivated crops (range = 0–93%, 50^th^ percentile = 0.4) was one of the most important in our AIC-best model. When cultivated crops were not present, probability of bear incident report occurrence exceeded 0.5 at 77% deciduous forest cover. With 11% cultivated crop cover, probability of bear incident report occurrence exceeded 0.5 at 68% deciduous forest cover. Additionally, primary road densities had to be 58% greater at low levels of deciduous forest cover (i.e., <11%) than at high levels (i.e., >43%) for probability of bear incident report occurrence to exceed 0.5 for both regions.

**Table 3:** Best model parameter coefficients.

Independent Variables	β^a^	LCL^b^	UCL^c^
Ecological variables			
Percent NLCD^d^			
Deciduous forest	2.88	2.39	3.37
Woody wetlands	2.79	2.33	3.25
Open water	1.54	1.32	1.76
Evergreen forest	1.44	1.18	1.70
Mixed forest	1.22	1.03	1.41
Grassland-herbaceous	1.11	0.98	1.25
Emergent herbaceous wetlands	0.55	0.39	0.70
Barren land	0.42	0.33	0.51
Shrub-scrub	0.41	0.30	0.52
Weighted NDVI^e^ mean	0.20	0.08	0.32
Anthropogenic variables			
Percent NLCD			
Cultivated crops	2.09	1.68	2.50
Pasture-hay	1.10	0.90	1.31
Developed open space	0.70	0.56	0.84
Developed high intensity	0.04	-0.09	0.17
Primary road density^f^	1.51	1.36	1.67
Secondary road density^g^	0.15	0.08	0.21
Region^h^			
Upper Peninsula	0.27	0.11	0.44
(Intercept)	-1.42	-1.38	-1.05

Independent variables in the AIC-best model describing the spatial relationship between landscape parameters (centered and scaled) and black bear incident report occurrences, Michigan, USA, 2003–2011.

^a^ β = coefficient estimates.

^b^ LCL = lower 95% confidence limits.

^c^ UCL = upper 95% confidence limits.

^d^ NLCD = National Land Cover Database; percent NLCD = percent area for each land cover.

^e^ NDVI = Normalized Difference Vegetation Index.

^f^ Primary road density = interstates, highways, and residential roads.

^g^ Secondary road density = roads that may be paved but have little traffic (e.g., park roads, two-track roads, etc.).

^h^ Region = categorical variable: reference region was Lower Peninsula.

**Table 4:** Summary of independent variables.

	Bear Incident Reports		Random Units	
Independent Variables	Mean	SD	Mean	SD
Ecological variables				
Percent NLCD^a^				
Deciduous forest	30.49	19.79	28.77	22.56
Woody wetlands	19.51	17.98	22.05	20.66
Open water	4.22	9.79	2.78	9.14
Evergreen forest	8.58	9.79	7.97	11.80
Mixed forest	7.30	7.37	6.22	7.97
Grassland-herbaceous	5.41	6.66	3.46	5.20
Emergent herbaceous wetlands	1.56	2.63	2.38	5.89
Barren land	0.48	1.61	0.46	3.06
Shrub-scrub	2.04	3.13	1.97	4.24
Weighted NDVI^b^ mean	197.92	19.79	198.52	17.74
Anthropogenic variables				
Percent NLCD				
Cultivated crops	6.47	11.74	11.46	19.99
Pasture-hay	3.77	7.28	5.35	9.29
Developed open space	5.62	4.88	4.09	4.57
Developed high intensity	0.35	1.51	0.22	1.39
Primary road density^c^	0.00	0.00	0.00	0.00
Secondary road density^d^	0.00	0.00	0.00	0.00

Summarized values (mean ± standard deviation [SD]) of all continuous independent variables by used (i.e., Bear Incident Reports) and random units within the dataset of black bear incident report occurrences, Michigan, USA, 2003–2011. Standardization of variables (centered and scaled) was not conducted for the purposes of this table.

^a^ NLCD = National Land Cover Database; percent NLCD = percent area for each land cover.

^b^ NDVI = Normalized Difference Vegetation Index.

^c^ Primary road density = interstates, highways, and residential roads (km/km^2^).

^d^ Secondary road density = roads that may be paved but have little traffic (e.g., park roads, two-track roads, etc.; km/km^2^).

**Fig 3:**
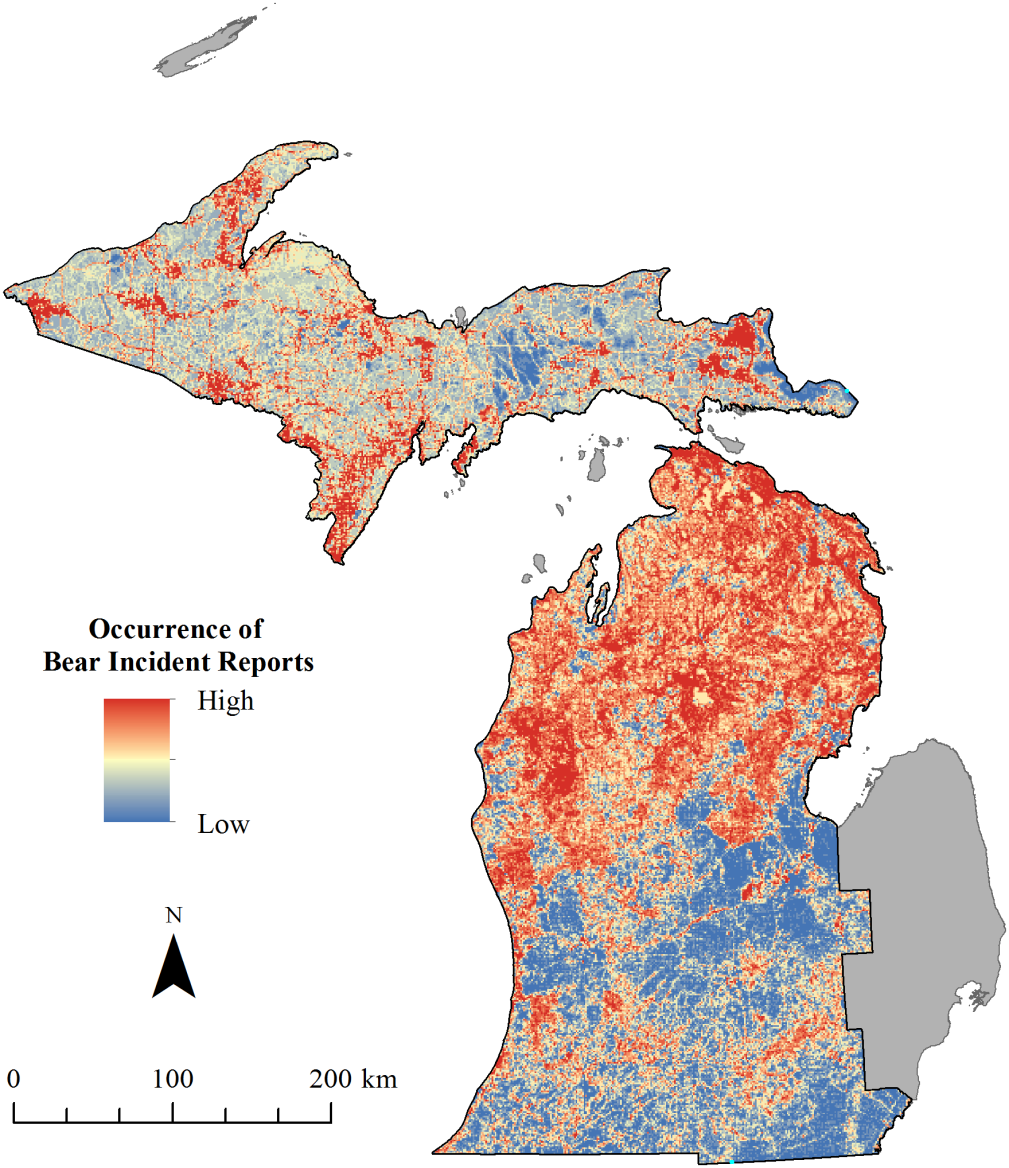
Relative distribution of the probability of black bear incident report occurrence in Michigan, USA. Based on black bear incident reports collected by Michigan Department of Natural Resources during 2003–2011. Solid gray areas were excluded from analysis as they contained no black bear incident reports and are outside the black bear population range.

The distribution of relative risk of bear incident report varied markedly throughout Michigan (Fig 3). Risk was relatively highest throughout the northern LP where there is a relatively medium density of bears in a fragmented landscape. The UP was mostly medium risk despite having a denser black bear population and a landscape that contained more forest cover. In contrast, southern Michigan, a highly agricultural landscape with few black bears, ranked relatively low for bear incident report risk with small patches of relatively greater risk.

## Discussion

According to our AIC-best model (Table 2) supported by model evaluation results, the amount of deciduous forest more strongly influenced the probability of bear incident report occurrence than other land covers in Michigan (Table 3). Evans et al. [65] also reported an increasing probability of human-black bear conflict occurrence with increasing percentage forest in exurban Connecticut, but only to a threshold (42%) after which probability declined. In an urban landscape in MT, Merkle et al. [66] found a negative association between probability of human-black bear interactions and distance to large forest patches (> 100 km^2^). We found that as the amount of deciduous forest cover increased, the probability of bear incident report occurrence increased across the diverse Michigan landscape. Though differences among study areas (e.g., human density, dominant land cover type) are evident, the relationship between bear incident report occurrence probabilities and forest cover are similar. Because black bears are forest obligates, bear densities may increase with increasing forest cover, due, in part, to greater natural food availability (e.g., spring ephemerals in vernal pools, tendency for some soft mast in summer, hard mast in fall; [67, 68]). Consequently, opportunities for bear incident reports in forested areas may increase, all other variables held constant.

We also observed a positive relationship between the probability of bear incident report occurrence and amount of cultivated crop cover. Black bears in North Carolina [69], northern LP of Michigan [50], and Colorado [70] used agricultural crops for food, especially when associated land-use activities occurred in or near preferred bear habitat. Baruch-Mordo et al. [70] also found agriculture-related conflicts were the most frequent human-black bear conflict type in Colorado. As opportunistic foragers, black bears may benefit from agricultural areas containing edible crops (e.g., corn, oats, sunflowers) because crop fields contain higher concentrations of food than forested areas [71]. Agricultural areas void of edible crops, however, may present high risk travel corridors for bears due to lack of cover [71]. Both scenarios may contribute to increased probability of bear incident reports depending on the spatial distribution and variability of resources. In fragmented habitat, bears exhibit greater space use which increases metabolic costs [26, 72] and the probability of encountering human activity. Our results suggest that the greatest relative probability of bear incident reports occurs in predominantly anthropogenic landscapes (e.g., greater road density, high crop cover) supporting relatively low bear densities. Supporting evidence from other studies suggests forest cover fragmented with agriculture or other anthropogenic activities presents an environment that likely facilitates human-bear interactions [73–75].

Primary roads had the second strongest effect of the anthropogenic landscape variables measured on bear incident report occurrence. Depending on the region’s primary mortality source (e.g., hunting or vehicular), road type (i.e., primary or secondary), dominant road activity type (e.g., vehicular travel, recreation access, hunting access), traffic volume (e.g., heavy hunting access during fall), and vehicle speed, bear movements and resource selection behaviors may be negatively influenced [28, 29, 34, 76]. Though bears have been documented to avoid paved highways [77], Reynolds-Hogland and Mitchell [28] suggest bears show greater avoidance of unpaved roads than paved roads. As hunting is the primary cause of black bear mortality in Michigan [78], bears may exhibit avoidance of unpaved roads in the fall to escape hunting pressure. Unpaved road avoidance is often accompanied by a risk tradeoff between potential road-related mortality sources and further increases in the risk of vehicular-collisions for bears by being in closer proximity to paved roads [34]. Bears may perceive paved roads as lower risk than unpaved roads because they are unable to predict vehicular-collisions when vehicles are traveling at higher speed limits. Further investigating the complex relationship between roads and bear movements would benefit wildlife management and the public by providing additional information to decrease bear-vehicle collisions.

Though our dataset consists only of bear incident reports and does not reflect confirmed bear incidents, our model selection and evaluation results remain highly relevant and useful for management. Our map can help wildlife managers identify areas of bear incident report occurrence, which they can use to help develop strategies aimed at reducing conflicts. Of particular interest, the southeast portion of the study area, where few bear incident reports occurred, had a high predicted relative probability of bear incident report occurrence. This may be because the landscape attributes of this area are similar to other areas of high bear incident report occurrence even though the black bear population density is lowest in the southern LP relative to the rest of Michigan [47]. Presuming the bear population increases in the southern LP and considering current landscape features, managers can use our model to predict areas of potential high bear incident report occurrence and to identify areas where greater educational efforts may be beneficial. Some aspects of human activities (e.g., agriculture) may contribute to the suitability of suboptimal habitat, and for black bears in the LP, this may facilitate the expansion of the population’s southern range [9, 69]. Assuming continued increases of the bear population in the northern LP [45], increasing occurrences of bear incident reports are likely.

Human-wildlife interactions occur in areas where human and wildlife activities overlap (e.g., as a result of rural expansion near or into forests; [66, 79]). With expanding human and large carnivore populations, managers can expect conflicts to not only continue, but also increase in frequency [80]. Understanding the spatial patterns of predicted bear incident reports can be especially vital for managers facing opposition from stakeholders to bear-control measures or when needing to prioritize areas for the reduction of bear incidents. Our modeling procedure can be adapted for use in other study areas and other wildlife species provided managers record human-wildlife interactions as spatially explicit occurrences. By combining field measurements and remote-sensing data, wildlife managers can map human-wildlife interactions statewide. Researchers and wildlife managers can use similar mapping techniques to assess locations of specific conflict types or to address human impacts on endangered species. Timely, appropriate, and effective resolution of conflicts generally results in greater public tolerance of increasing wildlife abundance and distribution within an anthropogenically-altered landscape [81, 82]. The efficacy of conflict resolution will only likely become more vital as human and wildlife populations continue to intermix, placing greater pressures on wildlife managers.

## Supporting Information

S1 Dataset Black bear incident report dataset. Dataset contains selected black bear incident reports received by Michigan Department of Natural Resources during 2003–2011 for the state of Michigan used in the analysis.
(XLSX)

S2 Dataset Independent dataset. Dataset contains selected black bear incident reports received by Michigan Department of Natural Resources during 2012–2015 for the state of Michigan used for the model evaluation.
(XLSX)
